# A challenging diagnosis of a nonsecretor plasma cell dyscrasia with pleomorphic plasmablastic morphology

**DOI:** 10.1002/ccr3.3260

**Published:** 2020-09-18

**Authors:** Stijn Van Landeghem, Sara Capiau, Jean‐Louis Bayart, Philip Vlummens, Jo Van Dorpe, Nadine Van Roy, Jan Philippé

**Affiliations:** ^1^ Department of Laboratory Medicine Ghent University Hospital Ghent Belgium; ^2^ Department of Clinical Hematology Ghent University Hospital Ghent Belgium; ^3^ Department of Pathology Ghent University Hospital Ghent Belgium; ^4^ CRIG Cancer Research Institute Ghent Ghent University Ghent Belgium; ^5^ Center for medical genetics biomolecular medicine and Cancer Research Institute Ghent (CRIG) Ghent University Ghent Belgium

**Keywords:** flow cytometry, morphology, multiple myeloma

## Abstract

This report highlights the importance of integrating clinical, radiological, genetic, and pathological laboratory findings to make a correct diagnosis especially with challenging and rare entities.

## Graphical abstract

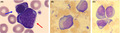

## 1 CASE REPORT

Differentiating plasmablastic lymphoma and plasmablastic myeloma is challenging due to overlapping morphological and immunophenotypical features. This case demonstrates the difficulty to distinguish a nonsecretor myeloma with plasmablastic morphology from a plasmablastic lymphoma and the necessity of a multidisciplinary approach to make an accurate diagnosis because the treatment protocols are different.

**Figure 1:**
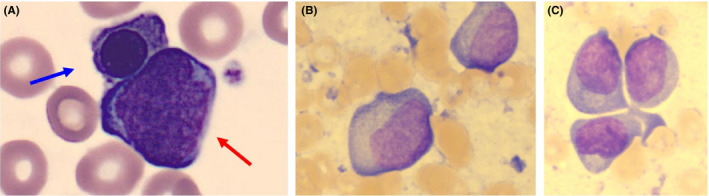
A: peripheral blood smear with an erythroblast (blue arrow) and a blastic cell (red arrow) (Wright‐Giemsa, ×1000), B and C: bone marrow smear with pleomorphic blastic cells with immature chromatin and a conspicuous nucleolus. Some blast cells display a distinctive perinuclear Golgi zone (C) (Wright‐Giemsa, ×1000)

A 70‐year‐old patient initially consulted his general physician because of a persisting anal abscess that did not respond to a two‐week antibiotic treatment with amoxicillin and clavulanic acid. A blood test to assess the patient's response to this infection unexpectedly showed thrombocytopenia, macrocytic anemia, and a normal white blood cell count with a leukoerythroblastic presentation (Table 1 and Figure 1A). Therefore, the patient was referred to the emergency department for further examination. Careful anamnesis revealed extensive weight loss (± 20 kg) during the last couple of months, increasing fatigue, decreased exercise tolerance, and right hypochondrial discomfort. Additionally, the patient reported no night sweats. Further physical examination revealed hepatomegaly, which was later confirmed by ultrasound examination. No lymphadenopathy nor other physical irregularities could be observed. The patient's medical history included hypercholesterolemia and poorly controlled type 2 diabetes. His previous blood test, performed three months earlier, did not show any hematological abnormalities (Table 1) and a negative HIV status. Based on these preliminary findings, the initial differential diagnosis included acute leukemia, myelodysplastic syndrome (MDS), and myelofibrosis.

**Table 1:** Laboratory findings of the peripheral blood examination at diagnosis and from the control blood test performed three months earlier

	At diagnosis	3 months earlier	Reference value
RBC (10^6^/µL)	2.53	3.80	4.25‐5.63
MCV (fL)	103.2	100	82.3‐96.4
Hemoglobin (g/dL)	8.9	13.4	12.9‐17.3
Erythroblasts (/µL)	190	0	<150
Platelets (10^3^/µL)	30	179	149‐319
WBC (10^3^/µL)	5.09	5.1	3.65‐9.30
Neutrophils (%)	52.1	41.0	38.9‐74.9
Lymphocytes (%)	36.0	51.3	16.1‐46.9
Monocytes (%)	6.9	5.7	4.6‐12.7
Eosinophils (%)	0.0	1.6	0.4‐5.0
Basophils (%)	0.5	0.4	0.2‐1.0
Plasma cells (%)	0.0	0	0
Immature granulocytes (%)	2.5	0	<1.0
Blasts (%)	2.0	0	0
Ca (mmol/L)	2.63	2.44	2.12‐2.62
CRP (mg/L)	15.7	3.3	<5.0
LDH (U/L)	386	136	105‐250
Creatinine (mg/dL)	0.99	0.77	0.72‐1.17
HbA1c (%)	11.8	8.8	4.0‐5.5
β_2_‐microglobulin (mg/L)	8.12	NA	1.09‐2.53

An examination of a bone marrow smear and trephine biopsy from the iliac crest was performed. The aspirate was stained with Wright‐Giemsa for cytomorphologic evaluation and showed a markedly decreased megakaryo‐, erythro‐, and myelopoiesis as well as the presence of a large population of blasts (75%) with a pleomorphic morphology. These cells displayed a moderate nuclear‐to‐cytoplasmic ratio, one or more prominent nucleoli, and a rim of basophilic cytoplasm. Although the morphology of most of these cells was compatible with monoblasts (50%) (Figure 1B), some cells displayed a perinuclear halo zone (25%) (Figure 1C), more reminiscent of plasmablasts. Due to the peculiar morphologic presentation of the blastic cells, the differential diagnosis was adjusted to the following entities: plasmablastic myeloma, plasmablastic lymphoma, plasma cell leukemia, and acute leukemia. Plasma cell leukemia was excluded since only 2% plasma cells were detected by flow cytometry, far below the 20% diagnostic criterium. Other laboratory findings (Table 1) included a mildly increased lactate dehydrogenase (LDH) and C‐reactive protein (CRP), no significant hypercalcemia, a normal kidney function, and a markedly elevated β_2_‐microglobulin level. IgG and IgA levels were mildly decreased, while IgM was not detectable. Serum‐free light chains were within normal values and displayed a normal κ/λ ratio. Moreover, serum electrophoresis and immunofixation could not detect a monoclonal fraction of IgG, IgA, IgM, IgD, IgE, kappa, or lambda. The latter experiments were repeated at 37°C to exclude precipitation of cryoglobulins, as well as with β‐mercaptoethanol pretreatment to rule out polymerization of a monoclonal M‐protein, all yielding the same result.

Flow cytometry was performed according to the EuroFlow protocols (Euroflow.org) to distinguish acute leukemia from a plasma cell neoplasm.^1^ The acute leukemia orientation tube (see details at euroflow.org) showed a large population with a distinctive high forward scatter (FSC) without a blastic phenotype: CD45‐, CD19_dim_, CD34‐, myeloperoxidase (MPO)‐, cytoplasmic CD3‐, cytoplasmic CD79a‐, and CD7‐. Subsequently, the plasma cell disorder panel confirmed the presence of neoplastic cells with a typical plasma cell immunophenotype: CD45‐, CD19_dim_, CD38_bright_, CD138+, CD28‐, and CD56‐. Interestingly, no cytoplasmic or surface light chain expression could be demonstrated in two independent analyses. Lack of CD56 expression along with the forward scatter properties allowed us to exclude a blastic plasmacytoid dendritic cell neoplasm.

**Figure 2:**
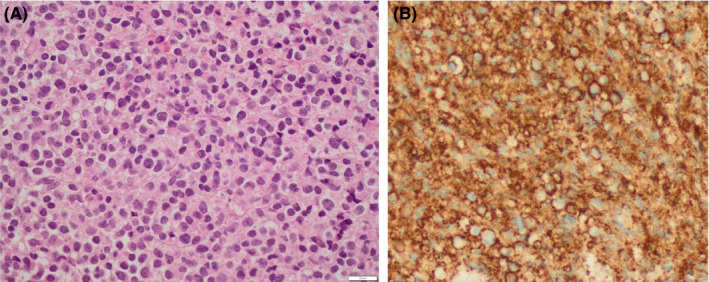
A. Bone marrow trephine biopsy with dense diffuse infiltrate consisting of atypical plasma cells (H&E, ×400), B. IHC of CD138 + plasma cells (×400)

Microscopic evaluation of the bone marrow trephine biopsy showed a limited presence of the three lineages and a striking invasion of neoplastic cells with plasmablastic aspect (Figure 2A). The reticulin staining corresponded to a myelofibrosis grade 1. Immunohistochemistry (IHC) confirmed bone marrow invasion by CD38 + and CD138 + neoplastic cells (Figure 2B). Additional staining showed MUM1 and cyclin D1 expression and absence of pan‐B markers (PAX5, CD79a, and CD20). Analogous to flow cytometry, no kappa or lambda expression could be demonstrated. IHC staining of IgG, IgA, and IgM showed no clear positive expression, with the exception of a weak reaction, possibly aspecific, for IgG in combination with a high background staining. The weak IgG expression at diagnosis was confirmed in a second trephine biopsy in follow‐up. Other analyzed markers (HHV‐8, LMP‐1, and MYC) were negative. Chromogenic in situ hybridization or EBV‐encoded RNA transcript (EBER) could not be performed since only decalcified material was available.

G‐banding analysis showed a complex karyotype. Fluorescence in situ hybridization (FISH) was performed on CD138 isolated cells and revealed the presence of a t(11;14)(q13;q32) without evidence for the presence of t(4;14), t(14;16), or MLL rearrangements. Shallow whole genome sequencing, also performed on DNA from CD138 isolated cells, showed the presence of segmental copy number gains and losses, as well as numerical chromosome aberrations. Multiplex polymerase chain reaction showed a clonal signal for the IgH and IgK light chains, thereby confirming the presence of a clonal B‐cell neoplasm. The t(11;14)(q13;q32) translocation is commonly observed in plasmablastic myeloma, whereas plasmablastic lymphoma is typically associated with a MYC‐rearrangement.

PET‐CT was compatible with the presentation of a disseminated myeloma invasion showing a diffuse invasion pattern of the bone marrow with multiple osteolytic lesions, without any extranodal localizations.

Although there are no definite criteria to differentiate nonsecretor plasmablastic myeloma from plasmablastic lymphoma, this case is most compatible with a nonsecretor myeloma based on the clinical presentation, disseminated bone marrow involvement, and lytic lesions without extranodal involvement, along with extensive laboratory investigations.

The patient was treated with a combination of velcade, endoxan, and dexamethasone (VCD), and further antibiotic treatment was sufficient to cure the anal abscess.^2^ After two VCD cycles, the bone marrow biopsy revealed a drop in plasmablastic cells from 75% to 15% and PET‐CT showed one residual metabolically active region in the right 4th rib. Two additional VCD cycles were given, and evaluation of a bone marrow biopsy showed a slightly elevated plasma cell count (5%‐10%) with atypical morphology. Despite the poor prognosis, the patient has a very good partial response (VGPR) after four VCD cycles and an autologous stem cell transplant is planned in the near future. The patient gave an informed consent for this case report.

## 2 DISCUSSION

This case demonstrates how challenging a correct interpretation of a bone marrow aspirate is when pleomorphic plasmablasts are present. Definite distinction between plasmablastic lymphoma and plasmablastic myeloma is difficult due to overlapping morphological and immunophenotypical features and the lack of distinctive immunophenotypic criteria.

Plasmablastic lymphoma was first described in 1997 by Delecluse *et al* as an aggressive lymphoma with neoplastic cells that have immunoblastic or plasmablastic appearance. Plasmablastic lymphoma predominantly occurs in patients with preexisting immunodeficiency, typically HIV, and is frequently located in extranodal sites, mainly the head and neck region or the gastrointestinal tract.^3^, ^4^ Later publications also described cases in immunocompetent patients.^5^, ^6^ The clinical image can mimic the characteristic presentation of a plasma cell myeloma with the presence of an M‐protein but a disseminated stage with bone marrow involvement is rarely seen at presentation.^5^, ^7^, ^8^, ^9^ Although plasmablastic lymphoma and plasmablastic myeloma show common features, a clear diagnosis is important because these two different disorders require different therapeutic protocols.^10^ The presence of an immunodeficiency or a positive EBER can be useful in the differential diagnosis. EBER is positive in 60%‐75% of the plasmablastic lymphoma cases, whereas latent membrane protein 1 (LMP1) is rarely expressed.^11^, ^12^ The immunophenotype of plasmablastic lymphoma consists of a characteristic plasma cell phenotype including CD138, CD38, and MUM1 positivity. Cytoplasmic immunoglobulins are commonly expressed, mostly IgG with either kappa or lambda light chain. Finally, some cases also express CD45, CD56, CD10, and CD79a, while cyclin D1 and B‐cell markers (CD20 and PAX5) are usually negative.^7^, ^9^, ^11^, ^13^

In contrast to the patient in this case report, the majority of plasmablastic lymphoma cases that resemble the clinical features of plasma cell myeloma have an HIV infection or suffer from another immunodeficiency. Secondly, the plasmablasts in this case have a plasma cell immunophenotype but are negative for CD45, CD79a, and CD56 which can be present, but not necessarily, in plasmablastic lymphoma. Interestingly, the production of a monoclonal immunoglobulin was only detected by intracellular IHC IgG staining. This intracellular IgG staining was only dimly positive in the bone marrow trephine biopsy at diagnosis and later confirmed in the first follow‐up biopsy. The lack of cytoplasmic light chain expression, both by flow cytometry and IHC, supports a defective immunoglobulin light chain production. The absence of cytoplasmic heavy or light chain expression has never been reported in plasmablastic lymphoma but can be observed in plasma cell myeloma variants.^14^ Approximately 1%‐3% of the plasma cell myelomas belong to a nonsecretor subtype and do not have detectable M‐protein in serum.^15^ A distinction is made within nonsecretor plasma cell myeloma variants between a defect in the secretion of a monoclonal Ig, that is, nonsecretor myeloma (85%), and a defect in the production, that is, nonproducer myeloma (15%). In the latter, no cytoplasmic heavy and light chains are detected.^16^, ^17^ The clinical presentation of a nonsecretor plasma cell myeloma, including the nonproducer variant, is similar to plasma cell myeloma except for a lower incidence of renal insufficiency due to the absence of an M‐protein, which is applicable in this case. The prognosis of plasma cell myeloma variants is similar to other types of plasma cell myeloma.^15^ The t(11;14)(q13;q32) and a complex karyotype are commonly detected in plasma cell myeloma but are also, although less frequently, described in plasmablastic lymphoma with HIV.^17^, ^18^ According to the genetic Mayo stratification, our patient has a standard risk based on the presence of a t(11;14).^19^ Unfavorable risk indicators in this case are, as stated by the international staging system, a high serum β_2_‐microglobulin level and a plasmablastic morphology, which underlines the impact of morphologic evaluation.^20^, ^21^ The latter is also highlighted in a recent case report of an aggressive presentation of a pleomorphic plasmablastic myeloma with the presence of an M‐protein.^22^

## 3 CONCLUSION

This case report describes the challenging diagnosis of a nonsecretor plasmablastic plasma cell myeloma and highlights the importance of remaining vigilant during morphological evaluation, regardless of the initially suggested diagnosis. Furthermore, it stresses how essential it is to integrate clinical, radiological, and laboratory findings to obtain all necessary information for a correct diagnosis, especially with challenging and rare entities. Although there are no comprehensive criteria to discriminate plasmablastic lymphoma from plasmablastic nonsecretor myeloma, distinction is important, as the treatment for these two diseases is different.

## CONFLICT OF INTEREST

None declared.

## AUTHOR CONTRIBUTIONS

Van Landeghem Stijn, Capiau Sara, Bayart Jean‐Louis, and Phillipé Jan analyzed and interpreted the peripheral blood, bone marrow morphology, and flow cytometry. Van Dorpe Jo analyzed and interpreted all immunohistological aspects. Van Roy Nadine analyzed and interpreted all genetic aspects. Vlummens Philip ensured the clinical anamnesis and follow‐up of the patient. Van Landeghem Stijn and Capiau Sara were major contributor in writing the manuscript. All authors read, revised, and approved the final manuscript.

## ETHICAL APPROVAL

The patient gave an informed consent for the publication of this case report.

## References

[1] Van Dongen JJM , Lhermitte L , Böttcher S , et al. EuroFlow antibody panels for standardized n‐dimensional flow cytometric immunophenotyping of normal, reactive and malignant leukocytes. Leukemia. 2012;26(9):1908‐1975. 22552007 10.1038/leu.2012.120 PMC3437410

[2] Moreau P , Hulin C , Macro M , et al.VTD is superior to VCD prior to intensive therapy in multiple myeloma: results of the prospective IFM2013‐04 trial. 2016. doi:10.1182/blood‐2016‐01. 10.1182/blood-2016-01-693580 27002117

[3] Tchernonog E , Faurie P , Coppo P , et al. Clinical characteristics and prognostic factors of plasmablastic lymphoma patients: Analysis of 135 patients from the LYSA group. Ann Oncol. 2017;28(4):843‐848. 28031174 10.1093/annonc/mdw684

[4] Delecluse HJ , Anagnostopoulos I , Dallenbach F , et al. Plasmablastic lymphomas of the oral cavity: A new entity associated with the human immunodeficiency virus infection. Blood. 1997;89(4):1413‐1420. 9028965

[5] Castillo JJ , Winer ES , Stachurski D , et al. HIV‐negative plasmablastic lymphoma: Not in the mouth. Clin Lymphoma, Myeloma Leuk. 2011;11(2):185‐189. 21575922 10.1016/j.clml.2011.03.008

[6] Teruya‐Feldstein J , Chiao E , Filippa DA , et al. CD20‐negative large‐cell lymphoma with plasmablastic features: A clinically heterogenous spectrum in both HIV‐positive and ‐negative patients. Ann Oncol. 2004;15(11):1673‐1679. 15520070 10.1093/annonc/mdh399

[7] Dong HY , Scadden DT , de Leval L , Tang Z , Isaacson PG , Harris NL . Plasmablastic Lymphoma in HIV‐Positive Patients. Am J Surg Pathol. 2005;29(12):1633‐1641. 16327436 10.1097/01.pas.0000173023.02724.1f

[8] Taddesse‐Heath L , Meloni‐Ehrig A , Scheerle J , Kelly JC , Jaffe ES , Taddesse‐Heath L . Plasmablastic lymphoma with MYC translocation: evidence for a common pathway in the generation of plasmablastic features HHS Public Access. Mod Pathol. 2010;23(7):991‐999. 20348882 10.1038/modpathol.2010.72 PMC6344124

[9] Meer S , Perner Y , McAlpine ED , Willem P . Extraoral plasmablastic lymphomas in a high human immunodeficiency virus endemic area. Histopathology. 2020;76(2):212‐221. 31361906 10.1111/his.13964

[10] Vega F , Chang C‐C , Medeiros LJ , et al. Plasmablastic lymphomas and plasmablastic plasma cell myelomas have nearly identical immunophenotypic profiles. Mod Pathol. 2005;18:806‐815. 15578069 10.1038/modpathol.3800355

[11] Castillo JJ , Bibas M , Miranda RN . The biology and treatment of plasmablastic lymphoma. Blood. 2015;125(15):2323‐2330. 25636338 10.1182/blood-2014-10-567479

[12] Morscio J , Dierickx D , Nijs J , et al. Clinicopathologic comparison of plasmablastic lymphoma in HIV‐positive, immunocompetent, and posttransplant patients: single‐center series of 25 cases and meta‐analysis of 277 reported cases. Am J Surg Pathol. 2014;38(7):875‐886. 24832164 10.1097/PAS.0000000000000234

[13] Montes‐Moreno S , Gonzalez‐Medina AR , Rodriguez‐Pinilla SM , et al. Aggressive large B‐cell lymphoma with plasma cell differentiation: Immunohistochemical characterization of plasmablastic lymphoma and diffuse large B‐cell lymphoma with partial plasmablastic phenotype. Haematologica. 2010;95(8):1342‐1349. 20418245 10.3324/haematol.2009.016113 PMC2913083

[14] Tominaga N , Katagiri S , Hamaguchi Y , et al. Plasma cell leukaemia of non‐producer type with missing light chain gene rearrangement. Br J Haematol. 1988;69(2):213‐218. 3134042 10.1111/j.1365-2141.1988.tb07624.x

[15] Kyle RA , Child JA , Anderson K , et al. Criteria for the classification of monoclonal gammopathies, multiple myeloma and related disorders: A report of the International Myeloma Working Group. Br J Haematol. 2003;121(5):749‐757. 12780789

[16] Drayson M , Tang LX , Drew R , Mead GP , Carr‐Smith H , Bradwell AR . Serum free light‐chain measurements for identifying and monitoring patients with nonsecretory multiple myeloma. Blood. 2001;97(9):2900‐2902. 11313287 10.1182/blood.v97.9.2900

[17] Dupuis MM , Tuchman SA . OncoTargets and Therapy Dovepress Non‐secretory multiple myeloma: from biology to clinical management. 2016. 10.2147/OTT.S122241 PMC5171196 28008276

[18] Gran C , Uttervall K , Borg Bruchfeld J , et al. Translocation (11;14) in newly diagnosed multiple myeloma, time to reclassify this standard risk chromosomal aberration? Eur J Haematol. 2019;103(6):588‐596. 31487754 10.1111/ejh.13325

[19] Mikhael JR , Dingli D , Roy V , et al. Management of newly diagnosed symptomatic multiple myeloma: Updated mayo stratification of myeloma and risk‐adapted therapy (msmart) consensus guidelines 2013. Mayo Clin Proc. 2013;88(4):360‐376. 23541011 10.1016/j.mayocp.2013.01.019

[20] Greipp PR , Miguel JS , Dune BGM , et al. International staging system for multiple myeloma. J Clin Oncol. 2005;23(15):3412‐3420. 15809451 10.1200/JCO.2005.04.242

[21] Greipp PR , Leong T , Bennett JM , et al. Plasmablastic morphology ‐ An independent prognostic factor with clinical and laboratory correlates: Eastern Cooperative Oncology Group (ECOG) myeloma trial E9486 report by the ECOG myeloma laboratory group. Blood. 1998;91(7):2501‐2507. 9516151

[22] Andres Suarez‐Londono J , Rohatgi A , Antoine‐Pepeljugoski C , Braunstein MJ . Aggressive presentation of plasmablastic myeloma. BMJ Case Rep. 2020;13(4):e234436. 10.1136/bcr-2020-234436 PMC7244288 32265213

